# Editorial: Ruminant Grazing Behavior: A Tool to Improve Product Quality and Ecosystem Services

**DOI:** 10.3389/fvets.2021.744200

**Published:** 2021-09-28

**Authors:** Thais Devincenzi, Mauro Coppa, Andrea Cabiddu

**Affiliations:** ^1^Instituto Nacional de Investigación Agropecuaria (INIA), Programa Producción de Carne y Lana, Estación Experimental INIA Tacuarembó, Tacuarembó, Uruguay; ^2^Independent Researcher at Université Clermont Auvergne, INRAE, VetAgro Sup, UMR1213 Herbivores, Saint-Genès-Champanelle, France; ^3^Agris Sardegna, Sardegna Agricoltura, Sassari, Italy

**Keywords:** pasture grazing management, cows, sheep—lamb, goats, milk

This Research Topic presents a series of original research and one review article that reveals the latest approaches to ruminant grazing behavior management associated with product quality and traceability. This collection embodies 12 original articles from eight countries, including Europe (Mediterranean, continental, and alpine regions) and North and South America. Articles were mainly focused on three axes: (i). pasture and grazing management and its relationship with ecosystem services, (ii). effect feeding behavior on animal's products, and (iii). genetics and grazing behavior.

Pasture and grazing management are some of the most powerful tools to manage and orient grazing behavior for both lambs and cattle, shown through studies conducted in controlled experiments or on field studies. In the studies published under this Research Topic, herbage allowance/structure, stocking rate, time allocation, and differences in grazing behavior between breeds were the investigated variables to set optimal management of both mono and multi-specific grasslands. Silva et al., in a subtropical environment, evaluated the ingestive behavior of young lambs considering the sward height. This study led to management recommendations for lamb production on tropical grasses under the ingestive behavior perspective, considering a maximum pasture height and a minimum lamb body weight to meet satisfactory intake parameters for optimizing lamb production. Poli et al. reviews the main available technologies for lamb production on pasture-based systems in subtropical regions. The proposed approaches are similar to classical pasture recommendations in other environments (i.e., controlling pasture availability, sward height, and structure), however, the authors identified a lack of studies on strategies to better manage herbage growth and minimize intense parasitic infections, which are common problem in subtropical regions.

Nicolao et al. evaluated if early-life dam-calf contact can influence post-weaning grazing behavior. The authors found that calves that experienced grazing with their dams until weaning immediately start to graze when turned out to pasture and expressed a grazing behavior more typical of adult cows than calves that were separated from their dams immediately after birth or to those that has not experienced previous dam contact. In temperate regions and on biodiverse pastures, Lind et al. described lamb performance and discussed opportunities and challenges for future sustainable sheep grazing on an island. The authors conclude that an adaptative management strategy must be adjusted to find an optimal compromise between animal production and restoring and conserving these environments, and therefore provide ecosystems services. Hamidi et al. monitored cow activity in a semi-natural grassland in Germany, under three levels of herbage allowance. At a lower herbage allowance, higher cattle activity is observed and at moderate allowances, a higher spatial distribution of cattle during grazing peaks is observed, with potential practical implications in the ecosystem. Finally, Noelle et al., in desert conditions, determined the optimal grazing time to avoid a second defoliation of swards and therefore indicates management targets to avoid degradation of grasslands in this fragile ecosystem. Pauler et al., in a controlled grazing experiment in the Swiss Alps, observed breed-specific differences in the behavior intake which can be used to improve pasture management and contribute to grassland conservation. These differences can be associated with allometry and anatomy of breeds adapted to graze nutrient-poor pastures. Having a general overview of the illustrated studies, their results highlighted that grazing behavior and management play a key role in pasture utilization and animal performances and welfare. Furthermore, grazing management and behavior can also be a powerful tool to drive ecosystem conservation and management, especially when exploited by adapted breeds, and more generally, to improve ecosystem services of pasture-based farming systems.

This Research Topic also explored the effect of grazing behavior and management on the characteristics of animal products, focusing on cow, ewe, and goat dairy products. Claps et al. in two case studies developed in the Italian mountain regions, provides information about milk quality, and discusses the relationship between grazing systems in biodiverse pastures and milk products with favorable characteristics for human nutrition, able to give high added value prices to dairy products. Studying milk from cows of breeds of different levels of specialization, Koczura et al. observed little differences in fatty acid composition according to breeds when grazing on the same pasture, suggesting that different grazing behaviors of adapted breeds could have consequences on dairy product characteristics. Molle et al. used fatty acids and n-alkane composition to trace feeding systems of ewes' milk. This study used mathematical models, such as Genetic Algorithms (GA) combined with Linear discriminant analysis (LDA), which allowed to discriminate products according to the amount of ingested pasture when using appropriate chemical markers. Complementary, Molle et al., used Fourier transformed mid-infrared spectroscopy (FT-MIR) to estimate milk FA composition, and the results obtained to discriminate milks sourced from dairy sheep rotationally grazing for 2, 4, or 6 h per day in Italian ryegrass or berseem clover. The authors conclude that is possible to discriminate milk from ewes grazing Grasses or Legumes using the GA-LDA of their FA profile. However, lower accuracy of the model was detected when they considered the different time allocations, probably also because of adaptation of grazing behavior according to the time restriction by ewes. At a genetics axis, Davis et al., explored the effect of breeding strategy on low input and organic production in dairy systems in the United Kingdom. The results of this study bring out the weakness of the breeding programs when animals face organic and low input production systems, in which grazing is the most common practice, and highlights the need to explore genotype x environment interactions to better guide breeding selection in this scenario. Thus, generally, grazing behavior and management can have an impact on diary product characteristics. Adapted breeds, exerting a different grazing selection than specialized dairy breeds, consume vegetation type, or patches which influence dairy product composition, particularly fatty acids. Differences in milk composition related to grazing management can be detectable for authentication purposes, with important perspectives for the valorization of dairy products issued from grazing systems.

Even if this Research Topic proposed important advancements in the research on grazing behavior and management as tools to improve product quality and ecosystem services, we can identify a lack of studies relating grazing behavior of ruminants on meat and wool products. Social studies and evaluation of environmental impacts and ecosystem services of grazing systems are just recently becoming more prominent as object of study, thus further research will be required in the future on these topics. Finally, new methodologies for monitoring feeding behavior and estimating herbage intake at pasture (especially on species rich and heterogeneous pastures) should be better explored in future research.

## Author Contributions

All authors listed have made a substantial, direct and intellectual contribution to the work, and approved it for publication.

## Conflict of Interest

The authors declare that the research was conducted in the absence of any commercial or financial relationships that could be construed as a potential conflict of interest.

## Publisher's Note

All claims expressed in this article are solely those of the authors and do not necessarily represent those of their affiliated organizations, or those of the publisher, the editors and the reviewers. Any product that may be evaluated in this article, or claim that may be made by its manufacturer, is not guaranteed or endorsed by the publisher.

